# Deceptive initial presentation of systemic DLBCL with CNS progression following Oligometabolic PET/CT: case report

**DOI:** 10.3389/fonc.2026.1735699

**Published:** 2026-03-02

**Authors:** Rui Yuan, Ya Yu, Qian Yang, Jun Chen, Kunlan Long, Peiyang Gao

**Affiliations:** Department of Critical Care Medicine, Hospital of Chengdu University of Traditional Chinese Medicine, Chengdu, China

**Keywords:** diffuse large B-cell lymphoma, immunohistochemistry, non-GCB subtype, PET/CT, secondary CNS lymphoma

## Abstract

We present a diagnostically challenging case of a 51-year-old woman with systemic diffuse large B-cell lymphoma (DLBCL) that progressed to secondary central nervous system (CNS) involvement. The initial presentation was notable for a whole-body PET/CT scan showing only subtle, diffuse fluorodeoxyglucose uptake in lymph nodes and bone marrow (SUVmax <5.0), below conventional thresholds for malignancy. Four months later, the patient developed an acute encephalopathic illness accompanied by multiorgan dysfunction and a severe hyperinflammatory state consistent with hemophagocytic lymphohistiocytosis. Hallmark laboratory features included refractory lactic acidosis, extreme hyperferritinemia, markedly elevated lactate dehydrogenase, and profound CD4+ lymphopenia. Cranial imaging revealed rapidly progressive, non−specific parenchymal lesions. A definitive diagnosis of the non−germinal center B−cell (non−GCB) subtype of DLBCL was secured via biopsy of a readily accessible facial lymph node—rather than high−risk brain biopsy—illustrating a pivotal diagnostic principle. This case highlights that unexplained persistent lactic acidosis, extreme hyperferritinemia, and even subthreshold PET/CT findings can be sentinel signs of an underlying aggressive lymphoma. It emphasizes the need for high clinical suspicion and the pursuit of safe, extracranial biopsy sites to enable early diagnosis and intervention in such diagnostically elusive cases.

## Introduction

Diffuse large B-cell lymphoma (DLBCL) is the most common subtype of aggressive non-Hodgkin lymphoma in adults, accounting for approximately 40% of all B-cell lymphomas. However, central nervous system (CNS) involvement at the time of initial diagnosis occurs in only about 5% of cases (1, 2). The CNS is an immune-privileged site, and due to the presence of the blood-brain barrier, DLBCL with CNS invasion typically progresses rapidly, responds poorly to treatment, and is associated with a high relapse rate. Patients with CNS involvement generally have a poor prognosis, with a median survival of approximately 3.4 months and a 2-year survival rate of 15% (3, 4). Clinical manifestations of CNS lymphoma are often non-specific and non-focal, including headache, altered mental status, seizures, cerebral edema, or ischemic-like lesions, which may mimic infectious or cerebrovascular conditions, thereby increasing the risk of misdiagnosis (5). Herein, we report a case of DLBCL that initially presented with subtle metabolic abnormalities on PET/CT—characterized by diffuse uptake in lymph nodes and bone marrow below conventional malignant SUV thresholds—which progressed to symptomatic CNS involvement within four months, with rapid intracranial lesion expansion within one week, and was ultimately diagnosed via extracranial lymph node biopsy.

## Case report

The patient was a 51-year-old Chinese woman admitted on August 15, 2025, due to fatigue and chest tightness. She had no significant prior medical history. Four months earlier (April 2, 2025), the patient sought medical attention for generalized fatigue accompanied by significant chest tightness and dyspnea. Since all initial investigations revealed no significant abnormalities, to evaluate metabolic abnormalities for the detection of potential malignancies, particularly occult lymphoproliferative disorders, whole-body PET/CT imaging was performed. In the neck, a localized area of increased density was observed in the fat space adjacent to the right masseter muscle, with mild FDG uptake (SUVmax 3.0). Bilateral submandibular and upper cervical lymph nodes were present, retaining visible hilar structures, with the largest measuring approximately 9 mm in short diameter and showing mild uptake (SUVmax 3.1, left side). In the thorax, a focal area of mildly increased uptake was noted in the outer quadrant of the left breast (SUVmax 1.9), without corresponding CT-detectable structural abnormality. Splenomegaly was identified. In the skeletal system, diffuse mild FDG uptake was observed in the bone marrow cavities of both axial and appendicular bones (SUVmax 4.7), without definitive osteolytic or sclerotic changes. On July 23, 2025, ultrasound revealed hypoechoic nodules in the skin and subcutaneous fat layers of both breasts and the right facial region, interpreted as enlarged lymph nodes. On August 8, 2025, laboratory tests showed elevated lactate (2.8 mmol/L) and lactate dehydrogenase (802 U/L).

**Figure 1:**
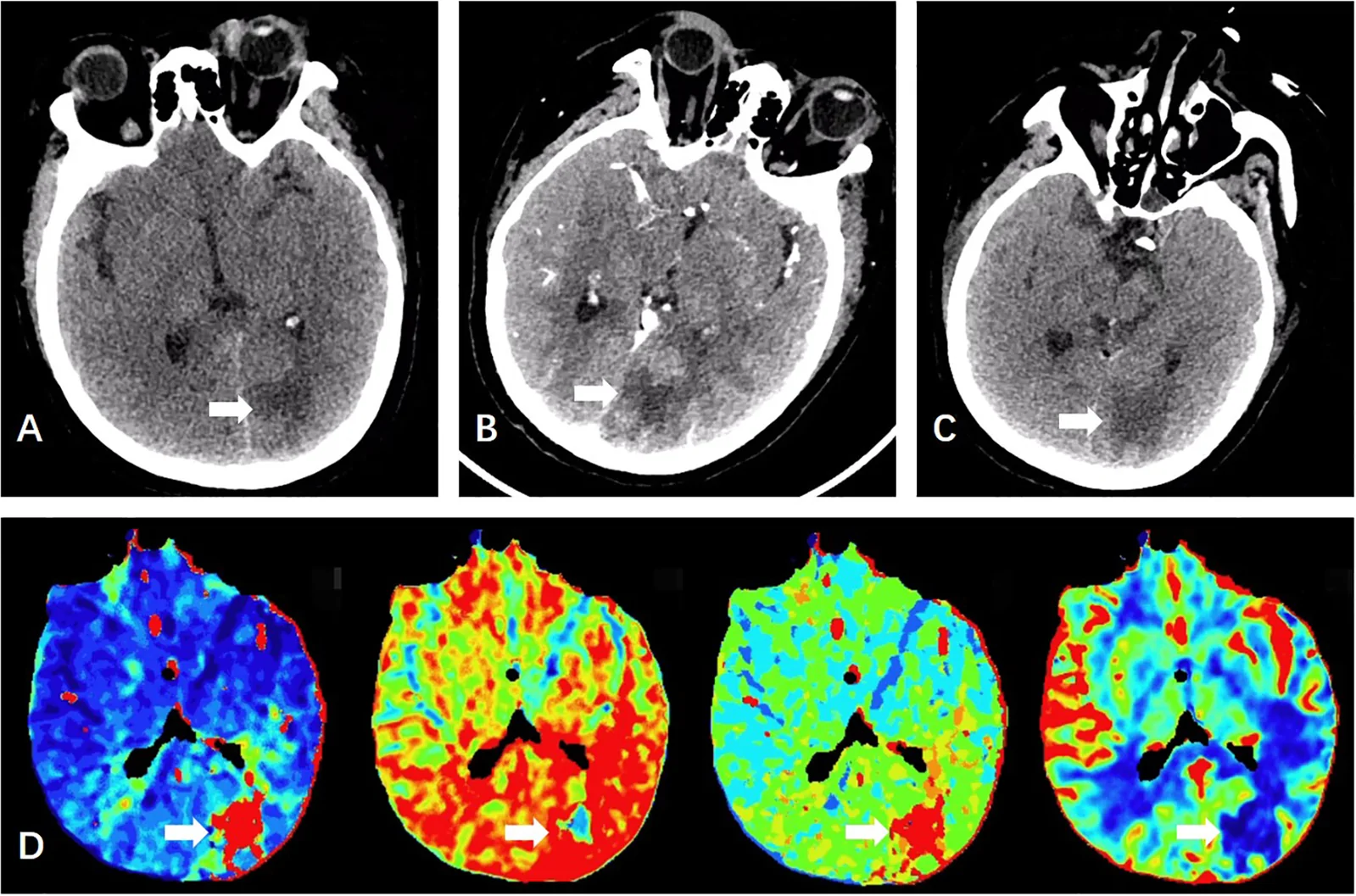
Initial non-contrast head CT (2025-08-19) reveals an irregular patchy hypodensity in the left parieto-occipital lobe **(A)**. Perfusion imaging shows reduced CBF, reduced CBV, reduced MTT, and prolonged TTP in the left parieto-occipital hypodense area: the volume with rCBF < 30% is approximately 0 ml, and the volume with Tmax > 6s is approximately 33 ml **(D)**. Contrast-enhanced head CT (2025-08-22) demonstrates the irregular patchy hypodensity in the left parieto-occipital lobe with ill-defined margins, showing no significant enhancement post-contrast **(B)**. A few small soft tissue nodules are noted in the left frontal and right submandibular subcutaneous regions, as well as bilaterally around the orbits, with the largest measuring approximately 9 mm in diameter. Follow-up head CT one week after treatment (2025-08-26) shows multiple irregular patchy hypodensities in the bilateral parieto-temporo-occipital lobes and the left frontal lobe. Compared to the previous scan, the involved areas have increased in extent, with some lesions being newly developed **(C)**.The lesion is shown as the white arrow in the picture.

At admission, vital signs were stable except for tachycardia (heart rate 128 beats per minute). Initial neurological examination revealed no focal deficits. Laboratory findings included leukopenia, lymphopenia, anemia, elevated inflammatory markers, lactic acidosis, increased lactate dehydrogenase, hepatic and renal dysfunction, heart failure, hyperuricemia, and hypertriglyceridemia (key results summarized in **Table 1**). On August 18, 2025, the patient abruptly developed altered mental status characterized by confusion, disorganized speech, restlessness, aggression, unresponsiveness to verbal stimuli, and responsiveness only to painful stimuli; limb muscle strength was graded 4–5. Concurrently, she developed hypotension requiring norepinephrine support and respiratory distress (oxygenation index 178.29 mmHg), necessitating transfer to the intensive care unit for supportive management. Non-contrast head CT demonstrated an irregular patchy hypodense lesion in the left parieto-occipital lobe (**Figure 1A**), with cerebral perfusion imaging revealing reduced perfusion in the same region (**Figure 1B**). Radiological findings suggested cerebral infarction; however, the extent and distribution of the lesion did not fully explain the severity of clinical symptoms. Given the constellation of systemic inflammation, neurological deterioration, and multiorgan dysfunction, differential diagnoses included sepsis, intracranial infection, hematologic malignancy, and intracranial space-occupying lesions. Empirical antimicrobial therapy (meropenem, vancomycin, ganciclovir) was initiated, along with mannitol for intracranial pressure reduction. Lumbar puncture was performed to obtain cerebrospinal fluid for microbiological and cytological evaluation, but results were negative.

**Table 1:** Key laboratory findings on admission, demonstrating hematological abnormalities, systemic inflammation, hepatic and renal dysfunction, elevated tumor markers, immune dysregulation, heart failure, and metabolic disturbances including hyperlactatemia, elevated lactate dehydrogenase, hyperuricemia, and hypertriglyceridemia.

Parameter	Value	Reference range	Parameter	Value	Reference range
WBC	2.73×10^9/L	3.5-9.5 ×10^9/L	CD4+/CD8+	1.04	1.5-2.5
NEUT#	1.55×10^9/L	1.8-6.3 ×10^9/L	TG	5.20 mmol/L	<1.7 mmol/L
LYMPH#	0.73×10^9/L	1.1-3.2 ×10^9/L	mHLA-DR	98.82%	90-100%
HGB	95 g/L	115–150 g/L	ANA	Positive (NuMA pattern, 1:100 titer)	Negative
PLT	76×10^9/L	100-300 ×10^9/L	ANCA	Weakly Positive	Negative
CRP	74.16 mg/L	0–5 mg/L	Viral PCR (EBV, CMV, HSV-II)	Negative	Negative
SAA	69.02 mg/L	0–10 mg/L	CSF NGS	Negative	Negative
TBIL	45.0 μmol/L	0-21 μmol/L	Cryptococcus Smear	Negative	Negative
DBIL	14.2 μmol/L	0-8 μmol/L	Cryptococcal Antigen	Negative	Negative
IBIL	30.8 μmol/L	0-17 μmol/L	FERRITIN	>2000.00 ng/mL	13–150 ng/mL
LDH	1002 U/L	120–250 U/L	NSE	27.44 ng/mL	0-16.3 ng/mL
Crea	99.2 μmol/L	41-73 μmol/L	CYFRA21-1	4.63 ng/mL	0-3.3 ng/mL
NT-proBNP	628.7 pg/mL	0–125 pg/mL	CA72-4	41.83 U/mL	0-6.9 U/mL
IL-6	18.11pg/mL	0–7 pg/mL	SCCA	10.000 ng/mL	0-2.5 ng/mL
CD3^-^CD16^+^CD56^+^	71 cells/μL	84–724 cells/μL	HE4	217.80 pmol/L	<74.3 pmol/L
CD3+	543 cells/μL	723–2737 cells/μL	ProGRP	125.30 pg/mL	<65.7 pg/mL
CD4+	252 cells/μL	404–1612 cells/μL	Thalassemia Gene	Negative	Negative
CD8+	243 cells/μL	220–1129 cells/μL	Abnormal Cell Morphology	Anisocytosis with Poikilocytosis	–

The patient was ultimately diagnosed with DLBCL.

Infectious etiologies were subsequently excluded. Following osmotic therapy, the patient’s mental status evolved from altered content of consciousness to predominantly impaired level of consciousness, manifesting as drowsiness, partial responsiveness to questions when aroused, occasional incoherent speech, and ability to follow simple commands. The patient’s inflammatory markers gradually decreased; however, there was no significant improvement in circulatory and respiratory function. Hemodynamic support continued to require vasoactive agents to maintain blood pressure, and respiratory support remained dependent on non-invasive or high-flow oxygen therapy. Due to the patient’s unstable clinical condition, MRI was not considered feasible. Consequently, a contrast-enhanced CT scan was repeated, which revealed a low-density lesion in the left parieto-occipital region. This lesion showed no significant enhancement on contrast administration, with poorly defined margins. Additionally, several small subcutaneous soft tissue nodules were identified in the left frontal region, right mandibular area, periorbital regions, and subcutaneous tissues around both orbits, the largest measuring approximately 9 mm in diameter (**Figure 1B**).

**Figure 2:**
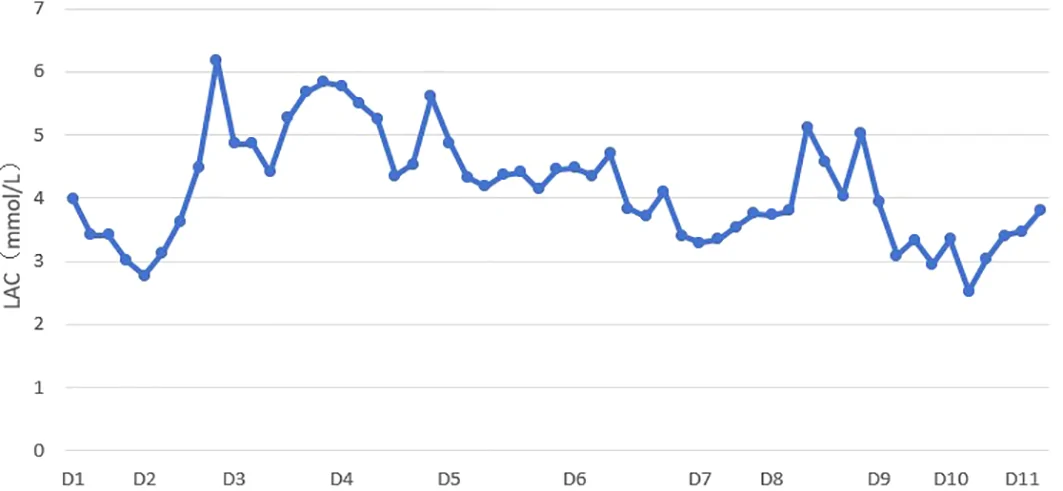
Line chart of the patient’s lactic acid levels. Serum lactate levels exhibited significant fluctuations during the disease course. Levels remained markedly elevated during the period of most severe impairment of consciousness and demonstrated a downward trend as the patient’s consciousness improved.

A comprehensive review of the patient’s prior medical history, presenting clinical features, and relevant laboratory and imaging findings revealed a previous history of elevated lactate and lactate dehydrogenase (LDH) levels. Previous PET/CT demonstrated several mildly FDG-avid lymph nodes in the bilateral submandibular and upper cervical regions, along with moderately increased bone marrow uptake in both the axial skeleton and appendicular bones, accompanied by splenomegaly. Although the SUVmax values were below the threshold typically associated with malignancy, these findings were still considered abnormal. Concurrent ultrasound examination revealed enlarged lymph nodes within the facial subcutaneous fat layer. Following the current admission, LDH levels remained elevated, and lactate levels persisted at high concentrations throughout the disease course (**Figure 2**), accompanied by markedly elevated serum ferritin. The patient developed sepsis-like systemic inflammation and acute heart failure in the absence of definitive evidence of severe infection or volume overload. Cerebral infarction was largely excluded based on neuroimaging findings. Given this constellation of clinical and laboratory abnormalities, hematological malignancy was strongly suspected. Consequently, we performed an extensive re-screening of the patient’s cervical and facial lymph nodes using ultrasonography. The largest lymph node in the left level IV cervical region measured approximately 11 × 8 × 6 mm, while the largest facial lymph node on the right side measured approximately 15 × 12 × 8 mm. Given its most superficial location and the lowest technical difficulty for biopsy, and after discussion with the general surgery team, a biopsy of the right-sided subcutaneous facial lymph node was decided upon.

**Figure 3:**
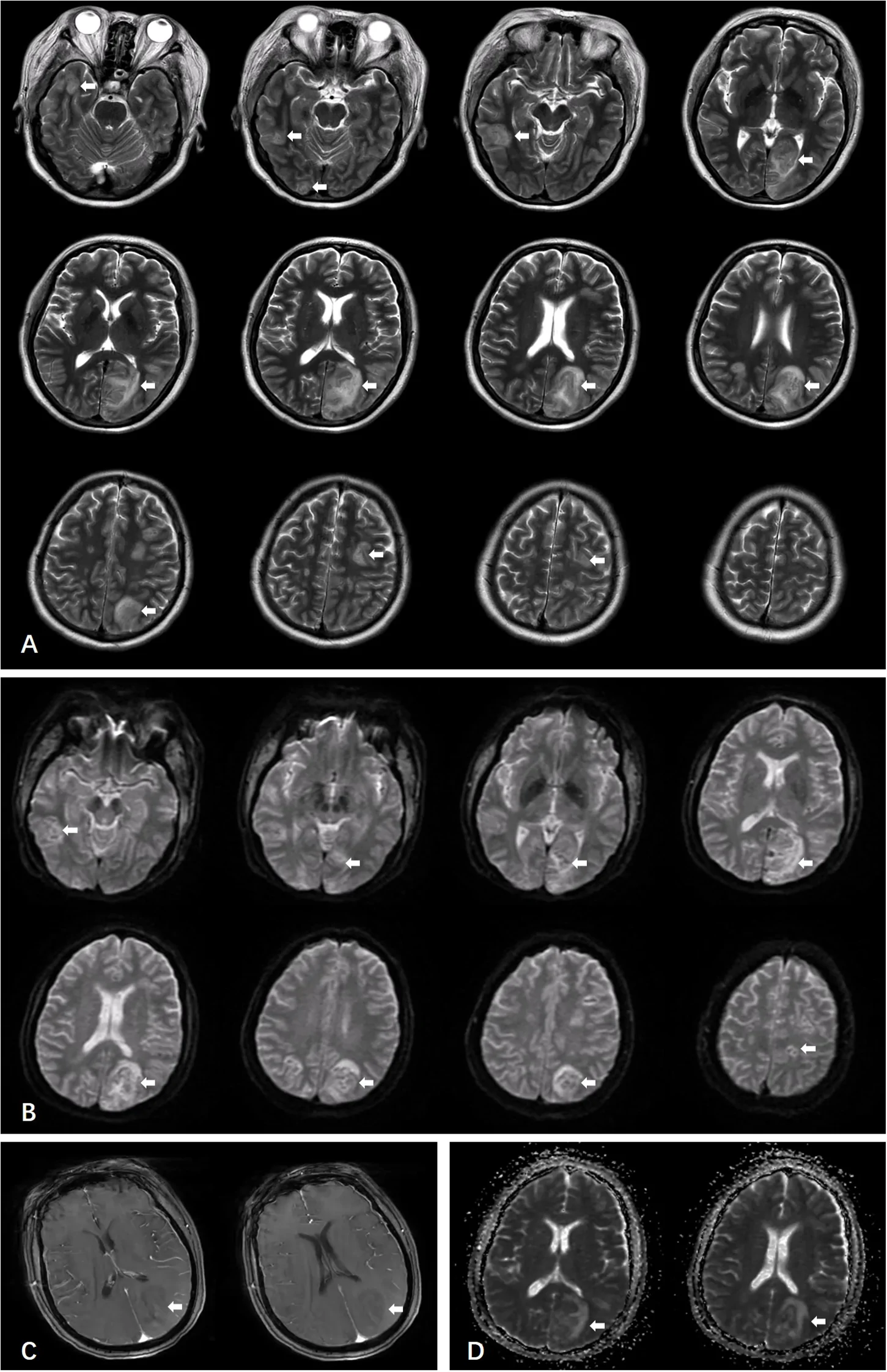
Multiple nodules and irregular patchy lesions showing long T1 and long T2 mixed signals are seen in the bilateral frontal lobes, parieto-occipital lobes, and centrum semiovale. The largest lesion, located in the left parieto-occipital lobe, measures approximately 5.0 × 2.8 cm **(A, B)**. The lesions appear slightly hyperintense on DWI, with a small area of hypotintensity in the left occipital lobe **(B)**. Post-contrast imaging shows heterogeneous mild enhancement of the lesions **(C)**. Multiple hypointense areas are observed on the ADC map **(D)**.The lesion is shown as the white arrow in the picture.

**Figure 4:**
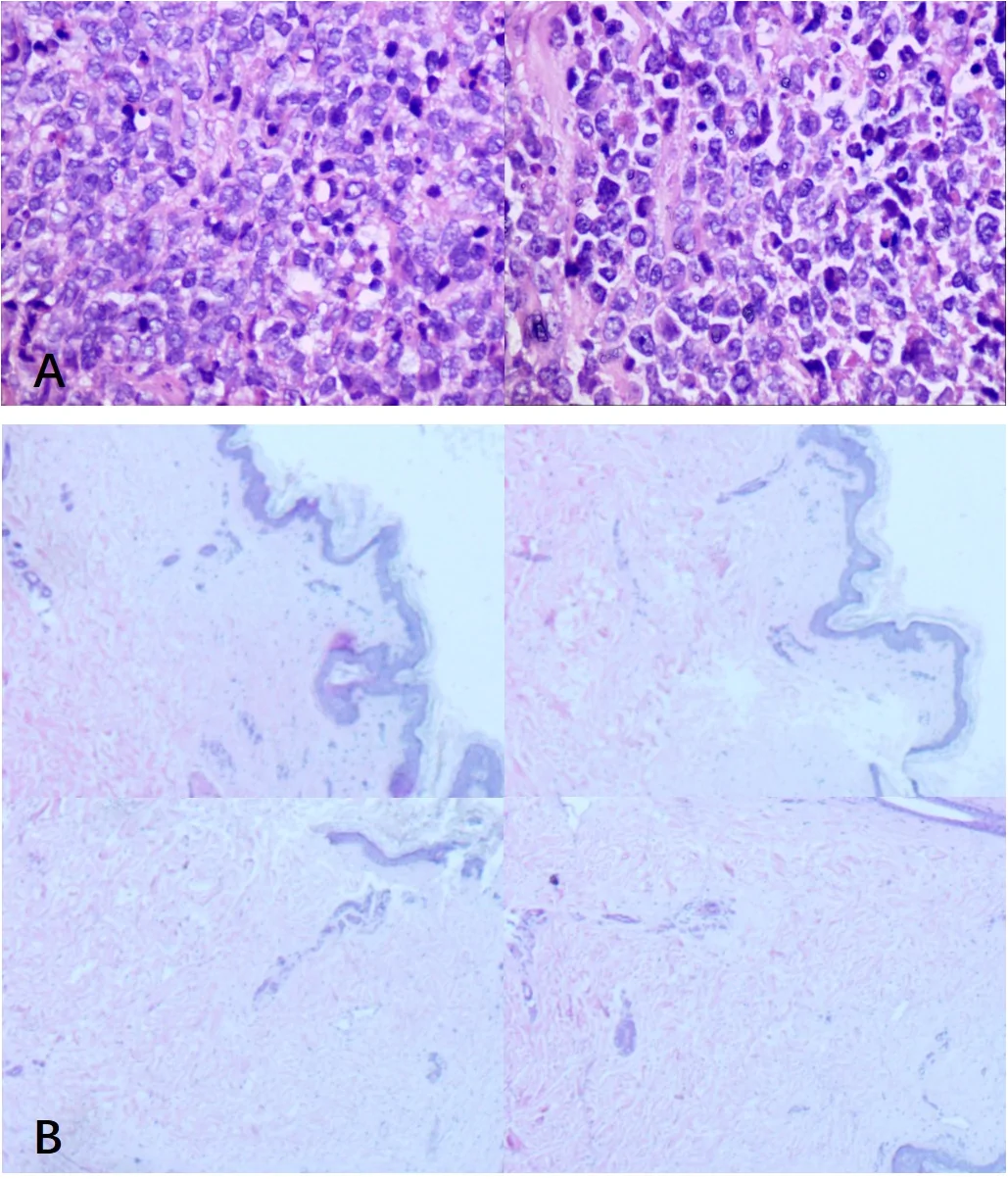
Lymphoproliferative disorder **(A)**. The nodal architecture is effaced by a diffuse infiltration of large atypical lymphocytes with prominent nucleoli and frequent mitotic figures. Morphology combined with immunohistochemical findings supports an aggressive B-cell lymphoma, consistent with diffuse large B-cell lymphoma, non-germinal center subtype. Immunohistochemical results: tumor cells are positive for Ki67 (approximately 60%), CD5 (+), CD20 (+), CD79a (+), Bcl-2 (approximately 90%), Bcl-6 (approximately 80%), MUM1 (approximately 60%), and c-Myc (approximately 10%); negative for CD3, CD10, and Cyclin D1; with CD21 showing follicular dendritic cell (FDC) network disruption. The histopathological examination of bilateral thigh skin specimens revealed well-preserved epidermal architecture with hyperkeratosis **(B)**. Both specimens exhibited occasional lymphocytic infiltration in the dermis accompanied by mild periadnexal fibrous hyperplasia. Additionally, the right thigh specimen demonstrated focal keratin cyst formation.

One week after initiation of supportive treatment (August 26, 2025), follow-up head CT revealed enlargement of the original lesion and emergence of new lesions (**Figures 1A, C**). The following day, once hemodynamically and respiratorily stabilized, urgent brain MRI was performed, which favored a neoplastic process (**Figure 3**). Concurrently, lymph node biopsy and immunohistochemical analysis confirmed diffuse large B-cell lymphoma (**Figure 4**).

A definitive diagnosis of non-germinal center B-cell-like (non-GCB) subtype DLBCL was established. To exclude intravascular large B-cell lymphoma (IVLBCL), two random skin biopsies were obtained, which revealed only epidermal hyperkeratosis without evidence of vascular or dermal lymphomatous infiltration (**Figure 4B**). The prior antibiotic therapy and mannitol administration for intracranial pressure reduction were maintained until August 29, 2025. The patient regained consciousness, demonstrated brief responsiveness, and was able to follow instructions and cooperate with motor tasks. However, due to financial constraints reported by the family, chemotherapy was declined, and the patient ultimately passed away.

## Discussion

This case illustrates the complex diagnostic journey of a patient with systemic diffuse large B-cell lymphoma (DLBCL) and secondary central nervous system (CNS) involvement, characterized by a highly deceptive initial presentation mimicking sepsis, intracranial infection, and cerebral infarction. The clinical course underscores the phenotypic heterogeneity and significant diagnostic challenges associated with aggressive lymphomas, particularly the non-germinal center B-cell (non-GCB) subtype.

First, the patient’s PET/CT scan performed four months prior to this admission had already indicated diffusely increased uptake in lymph nodes and bone marrow, yet these values did not reach the conventional thresholds for malignancy. This finding was the most misleading aspect in our search for the etiology and remained a point of confusion upon retrospective analysis, as it could have been mistaken for a peculiar subtype of low-metabolism DLBCL. It should be noted that there was a four-month interval between the PET/CT scan and the final diagnosis. Given the rapid progression of hematologic malignancies, the metabolic activity of the disease may have evolved during this period. However, due to the patient’s overall deteriorating condition, a repeat PET/CT was not feasible in the later stages. Therefore, we do not assert that this DLBCL was definitively FDG-negative; rather, we postulate that it may have presented with low metabolic activity at a very early stage of the disease. This atypical presentation was a key factor contributing to the diagnostic delay. A review of the literature revealed no prior reports of DLBCL demonstrating low uptake on ^18^F-FDG PET/CT, although a similar pattern was described in a case of recurrent breast implant-associated anaplastic large cell lymphoma, in which lymphoma was confirmed cytologically despite low FDG uptake on PET/CT (6). Additionally, one clinical study reported that among 5,980 detected lymphoma lesions, 52 were ^18^F-FDG-negative (including 18 nodal and 34 extranodal lesions). The underlying mechanism may be related to low expression of glycolysis markers in tumor cells, particularly glucose transporter 1 (GLUT1), whose expression strongly correlates with ^18^F-FDG SUVmax. Thus, low GLUT1 expression may be a key molecular mechanism leading to reduced ^18^F-FDG uptake in some lesions (7).

Second, following admission, the patient rapidly manifested a systemic inflammatory response accompanied by splenomegaly, trilineage cytopenia, hyperferritinemia, hypertriglyceridemia, and a marked reduction in natural killer (NK) cell counts—a constellation of findings consistent with hemophagocytic lymphohistiocytosis (HLH). This presentation strongly suggested an underlying lymphoproliferative disorder or neoplastic metabolic dysfunction. Retrospective analysis confirms this as classic DLBCL-associated HLH, a subtype of secondary/malignancy-associated HLH (MA-HLH). Among adults with secondary HLH, lymphoma is the most common trigger (accounting for 42%), with DLBCL representing one of the predominant subtypes within B-cell non-Hodgkin lymphoma (8, 9). Lymphoma cells themselves are the primary drivers of HLH pathogenesis. On one hand, they provide persistent antigenic stimulation; on the other, they may aberrantly secrete large quantities of pro-inflammatory cytokines such as interferon-gamma (IFN-γ). Together, these mechanisms lead to dysfunction or sustained hyperactivation of cytotoxic T lymphocytes and NK cells, impairing their ability to effectively clear activated macrophages. Subsequently, these activated CTLs and macrophages produce excessive cytokines, including IFN-γ, establishing a positive feedback loop known as a “cytokine storm,” which is the direct cause of tissue injury and multi-organ failure (10). Notably, inflammatory markers such as C-reactive protein (CRP) are typically significantly lower in HLH patients compared to those with sepsis, which can be attributed to the IFN-γ-driven nature of the cytokine storm. This explains why our patient exhibited severe multi-organ dysfunction and circulatory failure alongside only modest elevations in CRP, IL-6, and SAA (11). Concurrent with the activation of this exaggerated inflammatory response, counter-regulatory anti-inflammatory mechanisms are rapidly triggered, either sequentially or simultaneously. When the body’s overall response mechanisms become imbalanced, it can swiftly enter a state of persistent immunosuppression. In this case, this was evidenced by reductions in CD4^+^ T cells, CD3^-^ lymphocytes, and the CD4^+^/CD8^+^ ratio.

Moreover, molecular subtypes of DLBCL with a high risk of CNS invasion often exhibit a tumor microenvironment (TME) characterized by more pronounced immunosuppressive features. For instance, mutations in the IRF8 gene not only downregulate antigen presentation-related genes (such as CD74 and HLA-DM) but also lead to TME remodeling, manifesting as an increase in regulatory T cells (Tregs) and follicular helper T cells, alongside a decrease in CD4^+^ and CD8^+^ T cells as well as NK cells. This pattern is consistent with the patient’s observed reductions in CD4^+^ T cells, CD3^-^ lymphocytes, and the CD4^+^/CD8^+^ ratio (12, 13). This state not only heightens the risk of infection but also promotes the intrinsic progression of the disease. The CNS itself is a region with relatively weak immune surveillance. When the systemic immune system is compromised due to CD4^+^ T-cell dysfunction, the “sanctuary” effect behind the blood-brain barrier (BBB) becomes more pronounced, making tumor cells that have disseminated to the CNS more difficult to clear (14). The exhaustion or deficiency of CD4^+^ T cells can exacerbate CD8^+^ T-cell exhaustion and is accompanied by an increase in IL-17-producing CD8^+^ T cells, which have weak cytotoxicity, further undermining anti-tumor immunity (15).

Furthermore, studies have shown that HLH induces uncontrolled proliferation and activation of macrophages and lymphocytes, which is not confined to lymphoma lesions but is systemic (11). Activated macrophages infiltrate and exert phagocytic activity (hemophagocytosis) in organs such as the bone marrow, spleen, and lymph nodes. Concurrently, HLH triggers significant alterations in pathways related to systemic inflammation and bone marrow activation, including neutrophil extracellular trap (NET) formation and platelet activation. This leads to widespread mobilization and activation of bone marrow and immune cells, resulting in increased metabolic activity in these organs (9, 11). This mechanism is likely responsible for the refractory lactic acidosis and elevated lactate dehydrogenase observed in this patient. Under these circumstances, activated but non-neoplastic bone marrow/lymphoid tissue would present as diffuse, mild-to-moderate FDG uptake on whole-body PET/CT scans. This reduces the metabolic contrast between these inflammatory changes and actual lymphoma lesions. Consequently, small, relatively low-metabolic lymphoma foci may be obscured, while the specific high signal from larger, hypermetabolic lymphoma lesions may be “diluted” by the non-specific inflammatory activation signals. This ultimately complicates the interpretation of quantitative metrics such as SUVmax and total lesion glycolysis. Therefore, had a repeat PET/CT been performed after diagnosis for this patient, the metabolic findings from the lymphoma might have been superimposed on and difficult to distinguish from the background of systemic metabolic alterations.

Finally, the definitive diagnosis in this patient was achieved not through a high-risk brain biopsy but via a strategic biopsy of an accessible extracranial lesion—the right facial lymph node. This decision adhered to a key principle in neuro-oncology, enabling efficient and safe acquisition of a pathological diagnosis. Based on immunohistochemical results (CD20+, CD79a+, CD10-, Bcl-6+, MUM1+, Bcl-2 ~90%+, Ki-67 ~60%), a diagnosis of non-GCB DLBCL was confirmed. Integrating this with the PET/CT findings from four months earlier, which revealed lymph node and bone marrow involvement preceding CNS manifestations, and imaging demonstrating multiple subcutaneous nodules in the head and facial region suggesting systemic dissemination—ultimately corroborated by the facial lymph node biopsy—the overall clinical course was characterized by rapid systemic deterioration accompanied by CNS progression. This pattern aligns with the typical progression of systemic lymphoma with secondary CNS involvement. Therefore, the case is best classified as systemic DLBCL (non-GCB subtype) rather than primary CNS lymphoma. Regrettably, we were unable to perform any molecular testing, such as gene expression profiling, to further clarify the cell of origin.

Regarding the primary site, no cutaneous lesions were evident on physical examination. Random skin biopsies (**Figure 4B**) were performed to exclude intravascular large B-cell lymphoma (IVLBCL) or primary cutaneous lymphoma, revealing only epidermal hyperkeratosis without intravascular or dermal lymphomatous infiltration. Although prior PET/CT indicated diffuse FDG uptake in axial and appendicular bone marrow, and bone marrow involvement is known to increase the risk of CNS dissemination (16), primary bone marrow DLBCL is exceptionally rare and typically of the germinal center B-cell-like subtype, specifically originating from centrocytes (17), effectively ruling out a marrow origin. Additionally, the focal area of mild uptake in the outer quadrant of the left breast on prior PET/CT was subsequently confirmed by ultrasound to represent an enlarged lymph node, thus largely excluding a breast primary. In summary, this case is best classified as systemic DLBCL with secondary CNS involvement, demonstrating high CNS aggressiveness and marked neurotropism. The rapid progression of CNS lesions likely results from an interplay between intrinsic tumor cell driver mutations and an external, highly immunosuppressive microenvironment protected by the blood-brain barrier. This microenvironment was characterized by a state of profound immune suppression: CD4 ^+^ and CD8^+^ T cells were broadly exhausted; tumor-associated macrophages (TAMs) were polarized towards an M2-like phenotype, highly expressing immune checkpoint molecules such as PD-L1 and TIM-3 and secreting immunosuppressive factors like IL-10 and TGF-β; and immune checkpoint molecules (e.g., PD-1/PD-L1) were widely expressed on both tumor and myeloid cells, further impairing T-cell function. Additionally, specific spatial interactions between macrophages and vascular endothelial cells (BECs)—such as crosstalk between annexin A1-expressing endothelial cells and monocytes/macrophages expressing its formyl peptide receptors 1/2 and S100 calcium-binding protein A9—may have synergized with the immunosuppressive milieu to promote immune evasion, tumor proliferation, and resistance to conventional therapies (14, 18–20).

The fifth edition of the World Health Organization Classification of Haematolymphoid Tumours (WHO-HAEM5) introduces the novel category “Large B-cell lymphoma of immune-privileged sites (LBCL),” encompassing a group of aggressive B-cell lymphomas with shared biology, including DLBCL originating in the CNS, vitreous, and testes. These entities share morphological, immunophenotypic, and molecular features: typically CD10^-^, MUM1^+^, BCL6^+^, EBV-negative, with a propensity to involve other immune-privileged sites (21). Notably, some lymphomas arising outside classic immune-privileged sites (e.g., breast or skin) may exhibit overlapping characteristics, suggesting potential future expansion of this category. Intriguingly, the immunophenotype and aggressive clinical behavior in our patient closely align with features of LBCL. We hypothesize that this case may represent a unique form of systemic DLBCL originating outside classic immune-privileged sites yet displaying biological and clinical phenotypes similar to LBCL of immune-privileged sites.

Regarding management, per the National Comprehensive Cancer Network (NCCN) guidelines, induction therapy with rituximab, cyclophosphamide, doxorubicin, vincristine, and prednisone (R-CHOP) combined with high-dose methotrexate (HD-MTX) would be indicated (22). Chemotherapy could potentially alleviate neurological symptoms, the HLH-like syndrome, and the systemic inflammatory state. However, the prognosis remains poor. Real-world clinical studies indicate a median overall survival (OS) of approximately 11.4 months for patients with secondary CNS lymphoma; nevertheless, patients responsive to induction therapy who successfully undergo consolidative high-dose chemotherapy followed by autologous stem cell transplantation (HDT-ASCT) may achieve a 2-year survival rate exceeding 56%, with the possibility of several years of survival (23).

Notably, this report has significant limitations. We were unable to provide the original PET/CT images demonstrating FDG uptake below conventional malignant thresholds (SUVmax <5.0) in lymph nodes and bone marrow, which impairs readers’ direct assessment of the described ‘oligometabolic’ pattern. Furthermore, no metabolic imaging follow-up was conducted during the four-month interval between the initial PET/CT and the onset of CNS symptoms, precluding any dynamic characterization of metabolic activity changes in the lesions.

Nevertheless, this case also informs us that although high FDG uptake is typical for DLBCL, a minority of cases may present as ‘hypometabolic’ or exhibit ‘diffuse mild uptake.’ This phenomenon may be related to metabolic heterogeneity of the tumor cells (such as low GLUT1 expression levels) or masking of low-proliferation areas by an intense systemic inflammatory response. Therefore, in the differential diagnosis, even if PET/CT suggests low metabolic activity, the possibility of aggressive lymphoma should remain high on the list when accompanied by persistent elevation of systemic inflammatory markers, cytopenia, or neurological symptoms. For highly suspected cases, consideration should be given to short-term repeat neuroimaging (e.g., MRI) or whole-body PET/CT surveillance, alongside prompt pursuit of histopathological confirmation.

In conclusion, this case demonstrates that for any patient presenting with unexplained persistent lactic acidosis, markedly elevated LDH, and extreme hyperferritinemia (>2000 ng/mL), aggressive lymphoma must be prioritized in the differential diagnosis, even in the absence of definitive infectious evidence or typical imaging abnormalities. Any abnormal focus of uptake identified on PET/CT, regardless of subthreshold SUV values, warrants thorough evaluation when accompanied by non-specific systemic symptoms. Once lymphoma is suspected, priority should be given to identifying and biopsying accessible extranodal lesions (e.g., superficial lymph nodes or skin lesions) to facilitate timely diagnosis and avoid delays in treatment.

## Data Availability

The original contributions presented in the study are included in the article/supplementary material. Further inquiries can be directed to the corresponding authors.
