# Correction: Deceptive initial presentation of systemic DLBCL with CNS progression following oligometabolic PET/CT: case report

**DOI:** 10.3389/fonc.2026.2027139

**Published:** 2026-10-22

**Authors:** Frontiers Production Office

**Affiliations:** Frontiers Media SA, Lausanne, Switzerland

**Keywords:** diffuse large B-cell lymphoma, immunohistochemistry, non-GCB subtype, PET/CT, secondary CNS lymphoma

The funder “Chengdu University of Traditional Chinese Medicine Xinglin Scholar Program – Free Exploration Special Project, ZYTS2025012” to the authors was erroneously omitted.

The funder “Science and Technology Development Fund Project of the Hospital of Chengdu University of Traditional Chinese Medicine, 22XG01” to the authors was erroneously included.

The correct **Funding** statement reads: "The author(s) declared that financial support was received for this work and/or its publication. This study was supported by the Special Project for Traditional Chinese Medicine Research of Sichuan Provincial Administration of Traditional Chinese Medicine (Project Nos. 2021MS414, 25MSZX076 and 25MSZX276), and the Chengdu University of Traditional Chinese Medicine Xinglin Scholar Program – Free Exploration Special Project (Project No. ZYTS2025012)."

The original version of this article has been updated.

